# Expression of human endogenous retrovirus-K is strongly associated with the basal-like breast cancer phenotype

**DOI:** 10.1038/srep41960

**Published:** 2017-02-06

**Authors:** Gary L. Johanning, Gabriel G. Malouf, Xiaofeng Zheng, Francisco J. Esteva, John N. Weinstein, Feng Wang-Johanning, Xiaoping Su

**Affiliations:** 1 SRI International, Biosciences Division, 333 Ravenswood Ave, Menlo Park, CA, USA; 2 Department of Medical Oncology, Groupe Hospitalier Pitié-Salpêtrière, Université Pierre and Marie Curie (Paris VI), GRC5, ONCOTYPE-Uro, Institut Universitaire de Cancérologie, Assistance-Publique Hôpitaux de Paris, Paris, France; 3 Department of Bioinformatics and Computational Biology, The University of Texas MD Anderson Cancer Center, Houston, TX, USA; 4 Laura and Isaac Perlmutter Cancer Center, New York University Langone Medical Center, New York, NY, USA; 5 Department of Systems Biology, The University of Texas MD Anderson Cancer Center, Houston, TX 77030, USA

## Abstract

Human endogenous retroviruses (HERVs), which make up approximately 8% of the human genome, are overexpressed in some breast cancer cells and tissues but without regard to cancer subtype. We, therefore, analyzed TCGA RNA-Seq data to evaluate differences in expression of the HERV-K family in breast cancers of the various subtypes. Four HERV-K loci on different chromosomes were analyzed in basal, Her2E, LumA, and LumB breast cancer subtypes of 512 breast cancer patients with invasive ductal carcinoma (IDC). The results for all four loci showed higher HERV-K expression in the basal subtype, suggesting similar mechanisms of regulation regardless of locus. Expression of the HERV-K envelope gene (env) was highly significantly increased in basal tumors in comparison with the also-upregulated expression of other HERV-K genes. Analysis of reverse-phase protein array data indicated that increased expression of HERV-K is associated with decreased mutation of H-Ras (wild-type). Our results show elevation of HERV-K expression exclusively in the basal subtype of IDC breast cancer (as opposed to the other subtypes) and suggest HERV-K as a possible target for cancer vaccines or immunotherapy against this highly aggressive form of breast cancer.

Breast carcinoma is the most common cancer and leading cause of cancer death in women worldwide. It is expected that, in the United States, breast cancer will make up 29% of all new cancer cases among women in 2015, and it is currently the leading cause of cancer death among women aged 20 to 59^1^. To explore the molecular profiles of breast cancer, The Cancer Genome Atlas (TCGA) Network used an extensive set of technology platforms, including DNA copy number variation arrays, DNA methylation arrays, exome sequencing, messenger RNA arrays, microRNA sequencing, and reverse-phase protein arrays to characterize four main breast cancer subtypes: luminal A (LumA), luminal B (LumB), basal, and Her2-enriched (Her2E)^2^. They identified two new groups within the Her2-positive subclass, approximately half of them Her2E, the other half luminal.

The triple-negative breast cancer subtype (TNBC; defined by molecular markers) and the basal subtype (defined by histology) overlap extensively; both classes are predominately negative for estrogen receptor (ER), progesterone receptor (PR), and Her2^3^. Gene expression studies of basal tumors have shown overexpression of genes characteristic of breast basal-epithelial cells (positive staining for the basal cytokeratins 5/6 and 17), hence the nomenclature^4^. About 75–80% of TNBCs, defined by lack of expression of ER and PR and lack of overexpression of Her2, belong to the basal subtype. Basal breast cancer is one of the most virulent and deadly, but it is not well understood mechanistically^5^,^6^. It exhibits few targets for therapy^7^.

Endogenous retroviruses (ERVs) are remnants of ancient active retroviruses that infected germline cells, and these viruses are transmitted vertically through successive generations in a Mendelian fashion. ERVs have undergone repeated amplification and transposition to such an extent that human endogenous retroviruses (HERVs), which integrated into the human genome 30–40 million years ago, currently make up 8% of the human genome sequence^8^. Retroviruses, including HERVs, are composed of gag, pol, and env genes similar to those present in exogenous retroviruses such as human immunodeficiency virus (HIV) and human T cell leukemia virus (HTLV). They are bound on each end by long terminal repeats (LTRs), which serve as the retroviral promoters. The exact chromosomal locations of all endogenous retroviruses are currently under active investigation, since some do not correspond to gene annotations in common databases^9^. HERVs have been associated with a variety of human diseases and disorders, but they are also believed to have potential for benefit to the host. However, causal relationships with beneficial or harmful effects have yet to be firmly established^10^.

Human endogenous retrovirus type K [HERV-K(HML-2)] is a retrovirus that integrated into the primate genome as early as 55 million years ago^11^. Our previous investigations revealed that it is expressed in subtypes of breast cancer^12^,^13^, and that it provides a novel target for possible immunotherapy of breast cancer^14^,^15^,^16^. A number of ERVs were recently reported to be re-activated in tumors, and several showed overexpression in the tumors but low or undetectable expression in normal tissues^17^. However, it has remained unclear whether the various subtypes of breast cancer exhibit differential expression of HERV-K. To address that question, we have analyzed the large TCGA RNA-Seq database to evaluate HERV-K expression in breast cancer subclasses. Our results indicate that several families of HERV-K are overexpressed in the basal subtype.

## Results and Discussion

### HERV-K is overexpressed in basal breast cancer

In previous studies it was reported that HERV-K is overexpressed in breast cancer. However, the expression of HERV-K in subclasses of breast cancer has not been investigated. In the present study, we provide strong evidence that several loci of HERV-K are consistently overexpressed in the basal subclass of breast cancer. HERV-K expression has not previously been associated with basal breast cancer.

For the analysis, we searched the TCGA RNA-Seq database to evaluate expression of the HERV-K108 (7p22.1), HERV-K109 (6q14.1), HERV-K113 (19p12b), and HERV-K115 (8p23.1) loci in basal, Her2E, LumA, and LumB breast cancer subtypes. We analyzed the TCGA transcriptome data from 512 invasive ductal carcinoma (IDC) breast cancer patients, and their characteristics are shown in Supplementary Dataset 1 (which was downloaded from the Broad GDAC, based on TCGA data version 2016_01_28 for BRCA (http://firebrowse.org/?cohort=BRCA&download_dialog=true). The four HERV-K loci analyzed were chosen because they are the better studied insertions in the human genome^18^, and because they are located on several different chromosomes and thus are representative of HERV-K expression throughout the genome. In addition, those loci alone were the ones used to clone an infectious HERV-K(HML2) retrovirus by *in vitro* recombination to produce viral particles that could infect human cells and integrate with the exact signature of present day endogenous HERV-K^19^, thus indicating the relevance of these loci for establishment of infection by HERV-K viruses.

**Figure 1:**
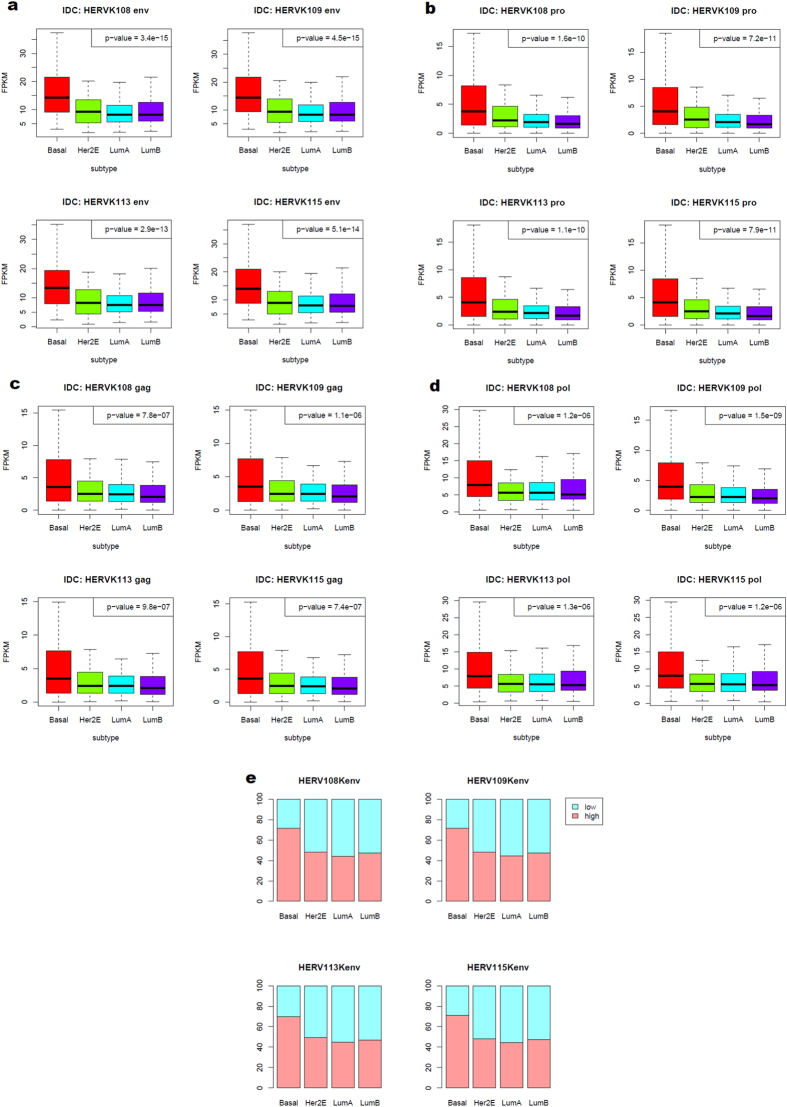
HERV-K mRNA expression in breast cancer patient tumors. HERV-K108, K109, K113, and K115 reference genome sequences and gene annotations were downloaded from NCBI GenBank (n = 512). Expression of HERV-K env (**a**), pro (**b**), gag (**c**), and pol (**d**) was evaluated. (**e**) HERV-K expression percent by subtype, expressed as the percentage of samples in each subtype above the FPKM median for all subtypes.

The most striking finding was overexpression of HERV-K in the basal subtype, regardless of the locus (Fig. 1). There was approximately 1.7-fold as much HERV-K expression in basal breast cancer as in the other three major subtypes. The relative differences among the patient subtypes were very consistent when HERV-K loci were compared, suggesting that HERV-K expression may be upregulated in a similar fashion in basal breast tumors at a number of HERV-K integration sites. Expression of the HERV-K envelope gene (env) was highly significantly increased (Fig. 1a) in comparison with the upregulated expression of other HERV-K genes (Fig. 1b–d) in the basal subtype. Approximately 70% of basal breast cancer patients showed high expression of HERV-K, compared with 50% or less for the other subtypes (Fig. 1e). Although the Her2E subtype did show elevated HERV-K expression relative to LumA and LumB, the increase was very modest (approximately 1.3-fold increase) compared with the much larger increase in the basal subtype (approximately 2-fold increase) (Fig. 1a).

We chose the selected HERV-Ks from among other established loci for this gene^8^ for several reasons. First, we wished to survey HERV-K loci that were present at different chromosomal locations (preferably on different chromosomes): HERV-K108 is located on chromosome 7, HERV-K109 on chromosome 6, HERV-K113 on chromosome 19, and HERV-K115 on chromosome 8. We also wanted to select HERV-K proviruses that had some functional relevance in humans and that shared common traits. HERV-K113 is present in the genomes of roughly 20% of humans and has full-length open reading frames (ORFs) for all viral proteins^20^. Like HERV-K113, HERV-K115 is a full-length provirus, but it contains a base-pair deletion in the gag gene, making it unlikely that the pro and pol ORFs can be transcribed^21^. The gag gene of HERV-K109 can support viral particle production and infectivity^20^, and HERV-K108 has a functional env gene^22^. When the env ORFs for the 4 HERV-Ks were expressed from a human expression vector in living, nonpermeabilized HeLa cells, the HERV-K proteins were detected at the cell surface, the site where a functional env protein would be expected to localize^22^.

Our results raise the question of why expression of only the basal subtype of breast cancer shows increased HERV-K expression. A related question is the role of HERV-K in the etiology of basal breast cancer. Since basal breast cancer is more aggressive than other subtypes and has a poor prognosis, the increased expression of HERV-K from various locations throughout the genome could, in part, be driving the aggressiveness of that breast cancer subtype. Several genes have been proposed as drivers of the basal phenotype, including hyperactivated FOXM1, MYC, and HIF1-α (also known as ARNT)^2^, as well as genes associated with an embryonic mammary epithelial signature^23^, Sox2^24^, and HDAC1^25^. Basal breast cancers have a high rate of metastasis, and we have reported that serum HERV-K levels at the time of breast cancer diagnosis are predictive of metastasis^13^. It was recently reported that sequences derived from endogenous retroviruses are activated in cancer cells and provide novel regulatory elements that may restructure the human transcriptional landscape in cancer^26^. Although speculative, the activation of HERV-K in the genome of basal breast tumors may engage a set of signaling pathways associated with poor clinical outcomes. That possibility will need to be addressed in future studies.

We analyzed the four major subtypes of breast cancer, but another subtype called claudin-low has been identified^27^. This subtype is categorized as having decreased expression of tight-junctions related genes (claudin 3, 4, and 7) and increased mesenchymal and stem cell-like characteristics. Most claudin-low tumors were characterized as being either basal-like or normal-like by the Prediction Analysis of Microarray 50-gene classifier (PAM50), and most showed a TNBC phenotype. It will be of interest to determine whether HERV-K is uniquely elevated in claudin-low or basal subtypes.

### Expression of HERV-K in basal breast cancer is higher in tumors with wild-type H-Ras

**Figure 2:**
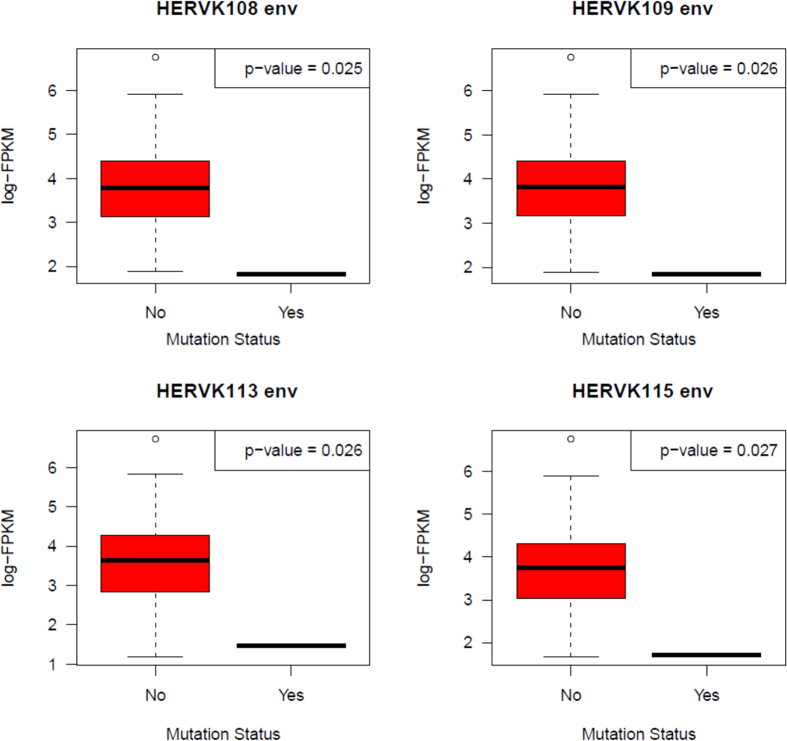
Association of HERV-K expression with H-Ras mutations in human breast tumors. H-Ras mutational status was downloaded from TCGA data version 2016_01_28 for BRCA (http://firebrowse.org/?cohort=BRCA&download_dialog=true) and used to analyze somatic mutations (n = 167).

Analysis of TCGA sequencing data indicated to us that increased expression of HERV-K is associated with decreased mutation of H-Ras (wild-type) (Fig. 2). We found that HERV-K targeting with a chimeric antigen receptor decreased expression of Ras (pan-Ras)^28^, and recently showed that Ras expression decreased as a result of HERV-K knockdown with an shRNA^29^, suggesting that HERV-K is necessary for full Ras expression. Wild-type Ras is capable of promoting development of cancers^30^, including breast cancer^31^, and basal breast cancer in particular^21^,^32^. There is a large body of data supporting the concept that wild-type Ras plays a critical role in cells that harbor Ras mutations^33^. Using TCGA data, it has been reported that over 30% of basal-like breast cancers display KRAS gene amplifications^34^, and an increase in genomic DNA copy numbers at the KRAS2 locus was reported in 9 of 16 human basal-like tumors^35^. Thus, another possible but as yet unexplored mechanism by which HERV-K could induce wild-type Ras overexpression is via effects on gene amplification. Our own HERV-K targeting data coupled with the TCGA data suggests that expression of HERV-K induces expression of wild-type unmutated Ras in basal breast cancer. We hypothesize that increased expression of wild-type H-Ras may lower the selective pressure to hyperactivate the Ras pathway through mutation.

### Correlation of HERV-K expression with expression of genes and proteins involved in cell signaling

**Figure 3:**
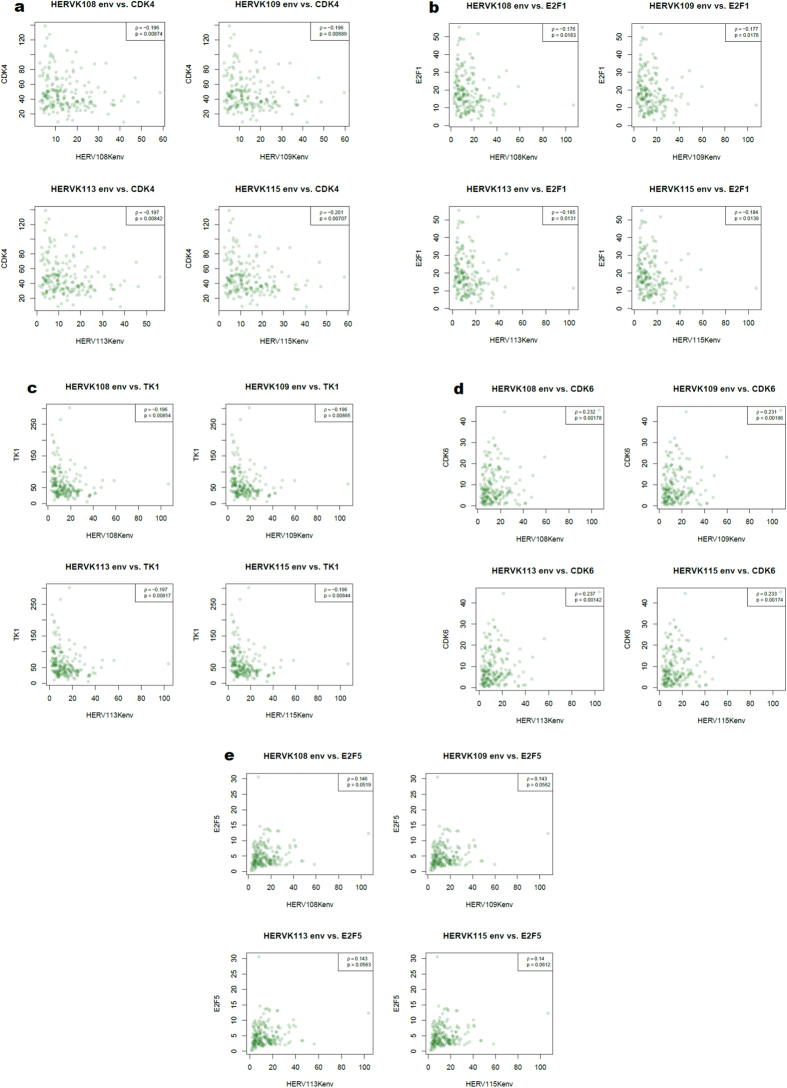
TCGA analysis of expression of HERV-K and cell signaling mRNAs in human basal breast tumors. Scatterplot showing associations between HERV-K expression and expression of cyclin-dependent kinase 4 (CDK4) (**a**) (n = 178; one outlier removed), E2F Transcription Factor 1 (E2F1) (**b**) (n = 179), thymidine kinase 1 (TK1) (**c**) (n = 179), CDK6 (**d**) (n = 179), and E2F5 (**e**) (n = 179). The units on the axes are FPKM.

**Figure 4:**
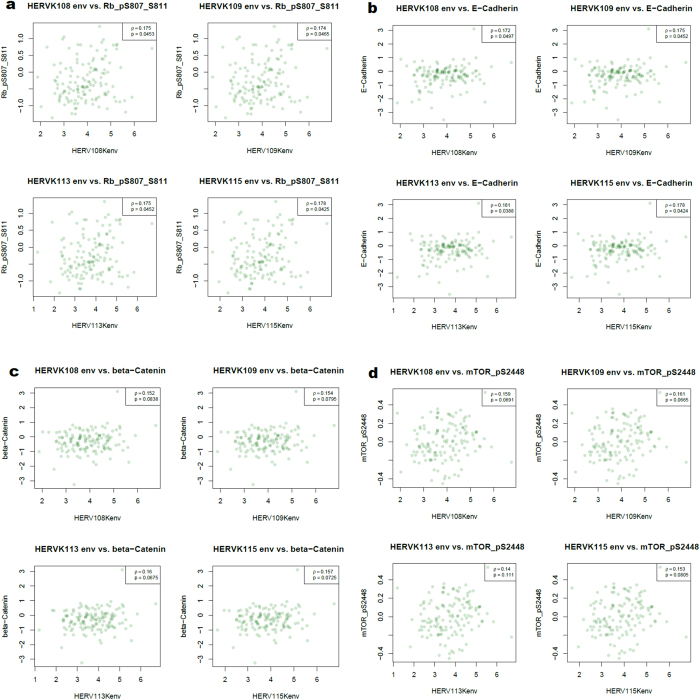
Association of basal breast tumor HERV-K protein expression with protein activities in signaling networks. RPPA data were used to analyze protein activities in signaling networks (http://gdac.broadinstitute.org/). The signaling proteins evaluated were p-Rb-s807/S811 (**a**), E-cadherin (**b**), β-catenin (**c**), and p-mTOR-s2448 (**d**). For each signaling protein analyzed, n = 131.

To identify signaling pathways that might be important in mediating the oncogenic action of HERV-K in basal breast cancer, we analyzed TCGA mRNA and reverse phase protein array (RPPA) expression data over the entire basal breast cancer gene set in relation to HERV-K expression. The genes and proteins discussed below are the ones that showed significant correlation with HERV-K in the IDC samples, or that approached significance. Expression levels of the cyclin-dependent kinase 4 (CDK4) (Fig. 3a), E2F Transcription Factor 1 (E2F1) (Fig. 3b), and thymidine kinase 1 (TK1) (Fig. 3c) genes were inversely correlated with HERV-K expression (P < 0.05). In contrast, expression levels of the CDK6 gene (Fig. 3d), the E2F5 gene (which inhibits cell cycle progression^36^) (Fig. 3e), and abundance of the retinoblastoma tumor suppressor protein (Rb) phosphorylated at sites S807/S811 (Fig. 4a) were positively correlated with HERV-K expression (P < 0.05). These findings suggest that HERV-K may play a role in Rb phosphorylation in breast cancer, since HERV-K expression associates with phosphorylation of this key tumor suppressor protein. In its unphosphorylated form, Rb inhibits expression of genes under the control of E2F transcription factors, including TK1, and Rb’s cell cycle suppressive function is inactivated by CDK phosphorylation. It is not clear why there is a differential association of CDK4 and CDK6 with HERV-K, but the maintenance of CDK6 (although not CDK4) activity has been reported to block differentiation^37^. In addition, a recent study in colon cancer cells revealed that shRNA knockdown of CDK6, but not CDK4, blocked Rb phosphorylation, and markedly inhibited cell proliferation^38^. Therefore, in breast cancer cells, high levels of CDK6 associated with HERV-K expression might increase Rb phosphorylation and promote its release from E2F transcription factors, thereby alleviating repression of S-phase genes, whose transcription is inhibited when E2F transcription factors are sequestered by unphosphorylated Rb protein. However, it should be noted that the p-values cited above, as well as those below, have not been corrected for multiple hypothesis testing across the array of genes. Hence, the associations suggested should be considered as the hypotheses for further exploration, rather than as the results of formal inference.

Overexpression of E2F1 has been reported to suppress transcription of the long terminal repeat (LTR) of human immunodeficiency virus type 1 (HIV-1), and the two elements responsible for repression were not consensus E2F1-binding sites^39^. If similar transcriptionally repressive, non-canonical E2F1 binding sites exist in the LTR of HERV-K, low expression of E2F1, and consequently its S-phase target gene TK-1, would lead to increased expression of HERV-K in breast cancer patients. Our recent report indicates that E2F1 functions as an upstream regulator of genes differentially expressed between breast cancer cell lines with and without knockdown of HERV-K env RNA^29^.

RPPA abundance levels of three additional proteins [E-cadherin (Fig. 4b), β-catenin (Fig. 4c) and p-mTOR-s2448 (Fig. 4d)] were positively correlated with HERV-K in basal breast cancer patients. The elevated levels of E-cadherin characteristic of cell-cell boundaries in normal epithelial cells are greatly decreased in breast cancer^40^. In addition, basal breast cancer is generally highly associated with epithelial-mesenchymal transition (EMT), and E-cadherin is down-regulated in EMT-related cancers. However, E-cadherin expression is rarely lost in basal breast cancer^41^,^42^, and E-cadherin mislocalization to the cytoplasm has been reported in human basal-like breast cancer cells^43^. E-cadherin is also subject to proteolytic cleavage, which results in shedding of its soluble ectodomain fragment, termed sEcad. Endogenous sEcad was reported to associate with Her1, Her2, and HER3 in Her2+ breast tumors, and to interact with Her1 in human TNBC specimens^44^. In the same study sEcad was shown to act with the Her ligand EGF to promote Her2+ breast cancer proliferation and migration, as well as TNBC invasion.

The possible association of HERV-K levels with abundance of β-catenin (P = 0.06–0.08) and p-mTOR-s2448 (P = 0.06–0.11) borders on significance, and the trend is seen for each of the four HERV-K loci studied. Alternative splicing of HERV-K transcripts leads to production of the novel nuclear oncoprotein Np9, whose increased accumulation upregulates β-catenin and promotes the growth of human myeloid and lymphoblastic leukemia cells^45^. Both cytosolic and nuclear β-catenin are increased in basal invasive breast cancers to a greater extent than in any other breast cancer subtype examined, and β-catenin is apparently associated with many features of basal cancer, including poor outcome^46^. Those results suggest that HERV-K–derived oncoproteins have the potential to upregulate a signaling pathway important to the progression of basal breast cancer.

Associations between HERV-K and mTOR phosphorylated at s2448 have not previously been reported. However, phosphorylated mTOR and proteins in its pathway have been reported to be downregulated in TNBC cells treated with mTOR inhibitors, and mTOR inhibitors blocked tumor growth by 77 to 99% in TNBC xenografts. That level of growth inhibition was significantly greater than seen with doxorubicin administration^47^. Other studies using TNBC cells and murine TNBC models have indicated that TNBCs are particularly sensitive to mTOR pathway inhibition^48^. The association between expression of HERV-K and phosphorylated mTOR may thus in part explain how increased HERV-K expression in basal breast cancer contributes to the pathology of the disease.

### Analysis of an additional dataset consisting predominately of invasive lobular carcinoma samples

**Figure 5:**
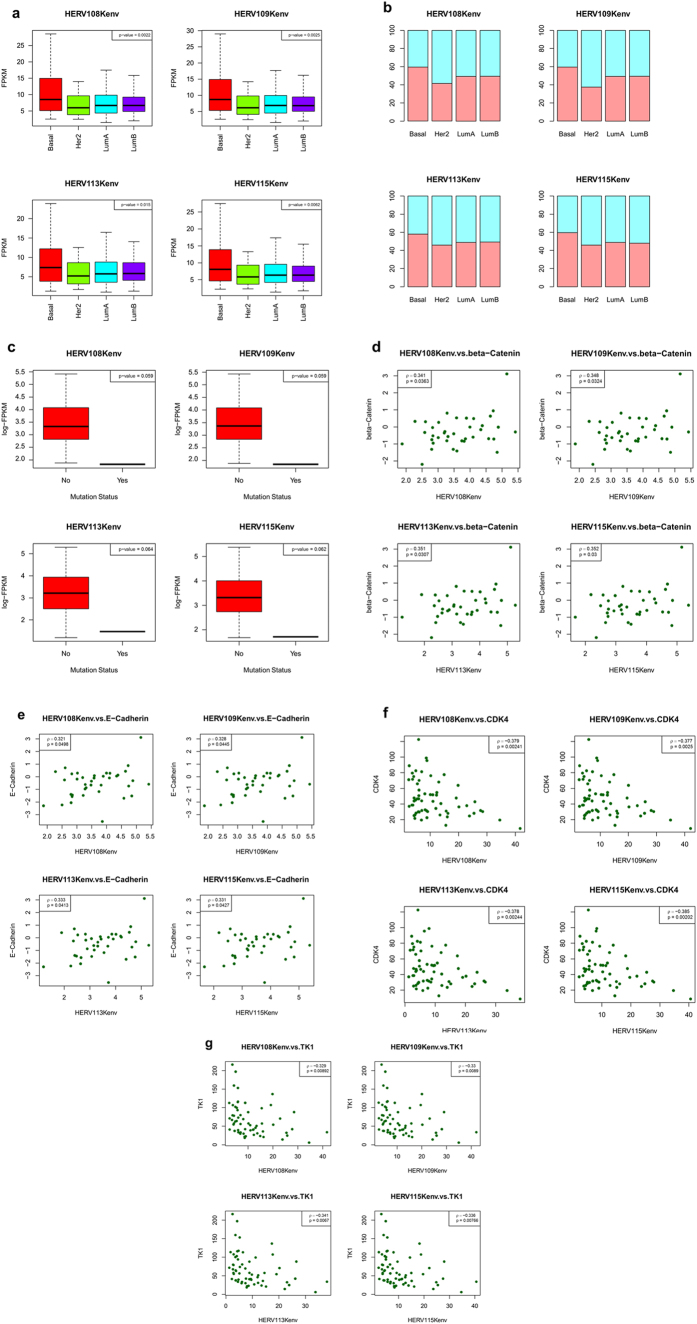
TCGA analysis of expression of HERV-K and cell signaling mRNAs in ILC/other human breast tumors. Expression of HERV-K env mRNA (**a**) and HERV-K expression percent by subtype, expressed as the percentage of samples in each subtype above the FPKM median for all subtypes (**b**) (for both (**a**) and (**b**): Basal, n = 62; Her2, n = 24; LumA, n = 315; LumB, n = 77). The association of increased expression of HERV-K with decreased mutation of wild-type H-Ras (**c**) (n = 53), and scatterplots showing associations between HERV-K expression and abundance of β-catenin (**d**) (n = 38), E-cadherin (**e**) (n = 38), CDK4 (**f**) (n = 62), and thymidine kinase 1 (TK1) (**e**) (n = 62).

Our original dataset was comprised of invasive ductal carcinoma (IDC) samples only. To determine whether the overexpression of HERV-K in basal breast cancer is unique to IDC, we queried an additional TCGA dataset that contained 10 IDC samples, 132 invasive lobular carcinoma (ILC) samples, 93 mixed samples, 159 non-annotated samples, and 119 other samples. Their characteristics are shown in Supplementary Dataset 2 (which was downloaded from TCGA data version 2016_01_28 for BRCA from Broad GDAC (http://firebrowse.org/?cohort=BRCA&download_dialog=true). Compared with the IDC samples, the remaining ILC/other samples showed increased expression of only the HERV-K env gene in basal breast cancer (Fig. 5a), but no increase in expression of the gag, pol, and pro genes. The HERV-K *env* gene in basal breast cancer in the ILC/other dataset was not as highly significantly overexpressed as it was in the IDC dataset (Fig. 5a,b). These additional data suggest that overexpression of HERV-K is more pronounced in IDC basal breast cancer patients than in ILC or other basal breast cancer types.

The association of increased expression of HERV-K with decreased mutation of wild-type H-Ras persisted in the ILC/other dataset (Fig. 5c), but that non-significant association was less robust than in the IDC dataset. The positive correlation of HERV-K expression with abundance of β-catenin (Fig. 5d) and E-cadherin (Fig. 5e), as well as inverse correlations with CDK4 (Fig. 5f) and TK1 (Fig. 5g), also held in the ILC/other dataset. However, the association between HERV-K expression and CDK6, E2F1, E2F5, and pRb S807/S811 was no longer apparent in the ILC/other dataset (data not presented). The analysis of this ILC/other dataset, which was predominately non-IDC, suggests that HERV-K associations with CDK6/Rb phosphorylation/E2F transcription factor release/derepression of S-phase genes, which was discussed in greater detail above, might be particularly relevant in IDC, whereas HERV-K effects on Ras mutation status, and on β-catenin, E-cadherin, CDK4, and TK1 abundance may be important in all breast cancer types.

These results give us a starting point to pursue mechanistic studies that are unique to HERV-K involvement in basal breast cancer patients with IDC, but not in those with ILC or other breast cancer types. It will be highly desirable to use these data to gain insight into pathway activities associated with HERV expression; that will be a high priority for future study.

### Summary

The current studies were undertaken to determine whether expression of HERV-K is activated in one or more breast cancer subtypes. The results show elevation of HERV-K expression exclusively in the basal subtype, perhaps associated with extremely poor prognosis and high frequencies of recurrence and metastasis. Because HERV-K is not observed in normal cells, it might be an excellent target for cancer vaccines against basal breast cancer, or for immunotherapy. In that regard, we have recently described therapeutic antibodies against HERV-K^14^. This study illustrates the value of TCGA data for discovery of novel biomarkers and for formulation of novel hypotheses, including hypotheses that may be useful in the attack on this particularly aggressive subtype of breast cancer.

## Methods

### RNA-Seq data analysis

Data were accessed from The Cancer Genome Atlas (TCGA). Breast invasive carcinoma (BRCA) RNA-Seq data (BAM files) and their related clinical data were obtained from Cancer Genomics Hub (CGHub, https://cghub.ucsc.edu/) and TCGA Data Portal (https://tcga-data.nci.nih.gov/tcga/). The paired-end FASTQ files for each sample were extracted from BAM files using bam2fastq (http://www.hudsonalpha.org/gsl/information/software/bam2fastq). A total of 512 invasive ductal carcinoma (IDC) samples were used for HERV-K expression profiling, which consisted of 117 basal, 53 Her2-enriched, 212 LumA, and 130 LumB by PAM50 classification (Supplementary Table 1). HERV-K reference sequences: Both the reference genome sequences and the gene annotations of HERV-K108, K109, K113, and K115 were downloaded from NCBI GenBank. Mapping/Alignment: The raw paired-end reads in FASTQ format were aligned to the human reference genome, GRCh37/hg19, using MOSAIK alignment software^49^. The raw paired-end reads in FASTQ format were also aligned to the human endogenous virus HERV-K108, K109, K113, and K115 reference genome sequences, respectively. MOSAIK works with paired-end reads from Illumina HiSeq, and uses both a hashing scheme and the Smith-Waterman algorithm to produce gapped optimal alignments and to map exon junction-spanning reads with a local alignment option for RNA-seq^49^. The resulting alignments were then saved as a standard bam file. The raw counts for each gene of both mRNAs and HERV-K genes were from RNA-seq. We then counted the mapped reads in genomic features such as genes (mRNAs) annotated in GENCODE15 and HERV-K genes [*env*, *pro*, *gag*, and *pol*] to generate the raw counts for each gene respectively using the HTSeq-count script distributed with the HTSeq package. We chose the “union” mode of HTSeq to mask the regions that overlapped between mRNAs and lncRNAs to overcome the issue of non-strand-specific RNA sequencing in the kit (TruSeq) used in TCGA data. FPKM calculation: We calculated the number of fragments per kilobase of non-overlapped exon per million fragments mapped (FPKM). Since the raw count data per gene was generated with the “union” mode in HTSeq, where the reads mapped to the overlapping regions between mRNAs and lncRNAs were not counted, the exon sequences for which overlap between mRNAs and lncRNAs exists were excluded when we calculated the gene lengths for both mRNAs and lncRNAs. A cutoff of median of overall HERV-K expression level was used to define low expression vs high expression. Ethics: The Ethics, Law and Policy Group was created by TCGA to identify and address critical ethical, legal and social questions faced by researchers and patients participating in the TCGA program. All ethics-related information can be found at TCGA Portal (http://cancergenome.nih.gov/abouttcga/policies/ethicslawpolicy).

### Reverse phase protein array (RPPA) data analysis

All of the RPPA data were downloaded from TCGA data version 2016_01_28 for BRCA from Broad GDAC (http://firebrowse.org/?cohort=BRCA&download_dialog=true).

## Additional Information

**How to cite this article**: Johanning, G. L. *et al*. Expression of human endogenous retrovirus-K is strongly associated with the basal-like breast cancer phenotype. *Sci. Rep.* **7**, 41960; doi: 10.1038/srep41960 (2017).

**Publisher's note:** Springer Nature remains neutral with regard to jurisdictional claims in published maps and institutional affiliations.

## Supplementary Material

Supplementary Dataset 1

Supplementary Dataset 2
